# Metabolic Characteristics of Hashimoto’s Thyroiditis Patients and the Role of Microelements and Diet in the Disease Management—An Overview

**DOI:** 10.3390/ijms23126580

**Published:** 2022-06-13

**Authors:** Aniceta A. Mikulska, Marta Karaźniewicz-Łada, Dorota Filipowicz, Marek Ruchała, Franciszek K. Główka

**Affiliations:** 1 Department of Physical Pharmacy and Pharmacokinetics, Poznan University of Medical Sciences, Rokietnicka 3, 60-806 Poznan, Poland; mkaraz@ump.edu.pl (M.K.-Ł.); glowka@ump.edu.pl (F.K.G.); 2 Doctoral School, Poznan University of Medical Sciences, Bukowska 70, 60-812 Poznan, Poland; 3 Department of Endocrinology, Metabolism and Internal Medicine, Poznan University of Medical Sciences, Przybyszewskiego 49, 60-355 Poznan, Poland; dorota.filipowicz123@gmail.com (D.F.); mruchala@ump.edu.pl (M.R.)

**Keywords:** autoimmune thyroiditis, hypothyroidism, diet, microelements, vitamins, supplements, metabolic disorders

## Abstract

Hashimoto’s thyroiditis (HT) is the most common autoimmune disease and the leading cause of hypothyroidism, in which damage to the thyroid gland occurs due to the infiltration of lymphocytes. It is characterized by increased levels of antibodies against thyroid peroxidase and thyroglobulin. In this review, we present the metabolic profile, the effectiveness of micronutrient supplementation and the impact of dietary management in patients with HT. For this current literature review, the databases PubMed, Cochrane, Medline and Embase were reviewed from the last ten years until March 2022. This article provides a comprehensive overview of recent randomized controlled trials, meta-analyses, and clinical trials. Many patients with HT, even in the euthyroid state, have excess body weight, metabolic disorders, and reduced quality of life. Due to frequent concomitant nutritional deficiencies, the role of vitamin D, iodine, selenium, magnesium, iron and vitamin B12 is currently debated. Several studies have underlined the benefits of vitamin D and selenium supplementation. There is still no specific diet recommended for patients with HT, but a protective effect of an anti-inflammatory diet rich in vitamins and minerals and low in animal foods has been suggested. There is insufficient evidence to support a gluten-free diet for all HT patients. Pharmacotherapy, along with appropriate nutrition and supplementation, are important elements of medical care for patients with HT. The abovementioned factors may decrease autoantibody levels, improve thyroid function, slow down the inflammatory process, maintain proper body weight, relieve symptoms, and prevent nutritional deficiencies and the development of metabolic disorders in patients with HT.

## 1. Introduction

Hashimoto’s thyroiditis (HT), also known as chronic lymphocytic thyroiditis or autoimmune thyroiditis (AIT), is a chronic inflammation of the thyroid gland with still incompletely defined etiopathogenesis. HT is the most common autoimmune disease and endocrine disorder, as well as the leading cause of hypothyroidism in iodine-sufficient countries [1,2,3]. The incidence of HT is a growing trend [4]. The diagnosis of HT is determined by biochemical (positive circulating thyroid autoantibodies) and imaging tests (hypoechogenic inhomogeneous thyroid structure in ultrasonography) with characteristic clinical features. HT is characterized by the production of anti-thyroid peroxidase antibodies (TPOAb) and anti-thyroglobulin antibodies (TgAb) [1,2,4,5,6,7]. Circulating TPOAb are found in about 90% of HT patients. TgAb are less sensitive (positive in about 60–80% of patients) and less specific than TPOAb [1,2,6]. As a result of inflammation, thyroid follicles are destroyed and replaced by small lymphocytes, which significantly reduces the echogenicity of the thyroid parenchyma in the ultrasound image [1]. HT predominantly affects the female population of any age, with an increased incidence in the middle-aged [2,6,7,8,9]. Women are about eight times more likely to develop HT than men. In addition, it is also more common in Whites and Asians than in African-Americans [1].

The pathogenesis of HT is related to lymphocytic intrathyroidal infiltration of T and B cells, especially of CD4+ Th1 and the production of antithyroid antibodies [1,2,4,7,10]. This leads to chronic inflammation and in consequence to fibrosis and gradual atrophy of the thyroid tissue [1,2,10]. HT is associated with various thyroid functional states ranging from euthyroid, subclinical to overt hypothyroidism [1]. The overt hypothyroidism is expressed by an increase in thyrotropin (TSH) and a decrease in free thyroid hormone levels [9].

HT negatively affects wellbeing and quality of life, because thyroid hormones are responsible for the rate of basal metabolism, metabolism of carbohydrates, proteins, and fats, in addition to thermogenesis. Clinical symptoms usually occur as a result of hypothyroidism and are characterized by local and systemic manifestations. HT affects various systems, including the cardiovascular, gastrointestinal, pulmonary, hematopoietic, reproductive, neuropsychiatric, as well as the skin. The symptoms of HT are non-specific (concentration problems, chronic fatigue, weakness, dry skin, changes in body weight and constipation) and they depend on the severity of HT [1,2,7,11]. Mood and anxiety disorders are also common in patients with HT. The cross-sectional study conducted by Yalcin et al. indicates that autoimmunity of the thyroid gland may have an impact on impaired health-related quality of life, depression and anxiety in euthyroid patients with HT independent of levothyroxine (LT4) substitution [12]. Some studies reported that even euthyroid HT patients have an increased predisposition to depression and anxiety disorders [13].

The exact mechanisms underlying AIT pathogenesis are not fully understood. Multiple factors from the external environment and the genetic background contribute to the pathogenesis of HT. These genetic, environmental, and existential factors provoke the immune system to produce antibodies to thyroid antigens [2,4,6,7,9,14,15,16,17,18]. The most important factors associated with this disease are summarized in Table 1.

Several genes have been involved in HT pathogenesis, including genes of the immune response (coded in the Human Leukocyte Antigen (HLA) complex) and thyroid function. Other immunoregulatory genes are involved in the development of HT, including the single nucleotide polymorphisms (SNPs) in cytotoxic T lymphocyte-associated antigen 4 (CTLA-4), protein tyrosine phosphatase non-receptor type 22 (PTPN22), and CD40 [2,4,6,7,18,19,20].

Among the environmental factors are insufficient or excessive iodine intake, infections, or the intake of certain medications [2,4,7,14,15,21]. Several of the currently used anticancer drugs, such as interferon-α, may cause autoimmune thyroid dysfunction [4,22]. The role of smoking and alcohol consumption in the etiopathogenesis of HT is still not clear. The data suggest that moderate alcohol consumption may protect against HT and the development of overt hypothyroidism [4,23,24]. Furthermore, some studies indicate that smoking decreases the levels of thyroid autoantibodies and the risk of hypothyroidism. However, the mechanism for these protective effects of smoking and drinking remains unclear and must be elucidated [4,24]. In recent years, the influence of stress on the development and course of HT has also been investigated. Some studies suggest that stress is involved in the pathogenesis of HT, while other evidence indicates that it has no effect [4,25]. A randomized controlled trial by Markomanolaki et al. showed that managing stress is also important in treating HT patients. After eight weeks of stress management intervention, patients demonstrated a reduction in TgAb titers, decreased levels of stress, depression, anxiety and improved lifestyle [26]. Additionally, the adequate levels of vitamin D and selenium (Se) may help prevent or delay the onset of HT [14,15,27,28]. Moreover, the risk of HT is increased in other autoimmune diseases [4,29].

**Table 1:** Genetic, environmental and existential factors associated with Hashimoto’s thyroiditis [2,4,6,7,9,17,18,20].

Genetic Factors	Environmental Factors	Existential Factors
Histocompatibility genes (HLA class I and II)	Iodine	Sex
Immunoregulatory genes (SNPs in HLA, CTLA-4, PTPN22, CD40 genes)	Medications (e.g., interferon-α, lithium, amiodarone)	Associated diseases (e.g., type 1 diabetes mellitus, pernicious anaemia, coeliac disease, myasthenia gravis)
Thyroid-specific genes	Infections (e.g., hepatitis C virus)	Age
Genes associated with thyroid peroxidase antibody synthesis	Smoking	Pregnancy
	Selenium	Down’s syndrome
	Vitamin D	Microbiome composition
	Alcohol	Familial aggregation
	Radiation Exposure	

The therapy of hypothyroidism as a result of HT is a daily, oral administration of synthetic thyroid hormone- levothyroxine, at a dosage of 1.6–1.8 micrograms per kilogram of body weight. The substitution therapy must be taken for life in order to maintain normal TSH levels [1,2,7]. In addition to the use of LT4, an appropriate diet and supplementation may be an important aspect of the treatment process. Se supplementation is associated with a significant decrease in TPOAb [30,31]. On the other hand, inadequate dietary Se intake may exacerbate HT [2,4,7]. Moreover, recent data demonstrated that vitamin D supplementation may have a beneficial effect on the course of HT [27,32,33].

In this review, based on the current literature, the influence of micronutrients and the effectiveness of selected types of diets in the treatment of HT were assessed. We investigated the relationship between micronutrient supplementation, including vitamin D and selenium, and Hashimoto’s thyroiditis. It has also not been established whether a gluten-free diet is necessary or whether any other diet may be of benefit in HT patients. Nutritional and supplementation guidelines for patients with Hashimoto’s thyroiditis to improve health and quality of life and reduce the incidence of complications have still not been developed. Moreover, there was a need to summarize the current knowledge about the effect of overweight or obesity on the risk of metabolic disorders and the role of oxidative stress in HT patients. Their influence on the course of Hashimoto’s thyroiditis has not been elucidated. We have tried to present a comprehensive view of these patients.

## 2. Methods

For this current literature review, the databases PubMed, Cochrane, Medline and Embase were searched for relevant studies from the last ten years until March 2022. The data covers the last 10 years when an intensive study of the Hashimoto’s thyroiditis population were conducted. We identified significant studies published in the interim that may affect our conclusions. This article provides a comprehensive overview of recent randomized controlled trials, meta-analyses, and clinical trials.

The databases were reviewed using the following phrases with words “Hashimoto’s thyroiditis”: diet, vitamin D, selenium, iodine, vitamin B12, iron, magnesium, metabolic disorders, oxidative stress, and microbiota.

Many patients with HT, even in the euthyroid state, have excess body weight, metabolic disorders, and reduced quality of life. Metabolic disturbances, improper diet, frequent nutritional deficiencies, chronic inflammation and the oxidative stress that occurs have a negative effect on the course of the disease. Therefore, the aim of the study was to summarize the current state of knowledge concerning metabolic characteristics of Hashimoto’s thyroiditis patients and the role of vitamins and minerals and diet in the management of the disease. HT is dependent on genetic and environmental factors, including nutrition or the use of supplementation, which can be modified. In this study, the role of the proper level of vitamin D and B12, iodine, selenium, magnesium and iron has been indicated. So far, no recommendations have been made regarding micronutrient supplementation. Moreover, a diet that may be of benefit to these patients has not been identified. Consequently, we have analyzed all nutritional and supplementation programs currently used in HT patients, as there is a need for guidelines for these patients.

## 3. Overweight or Obesity and Their Association with Metabolic Risk in HT

**Figure 1:**
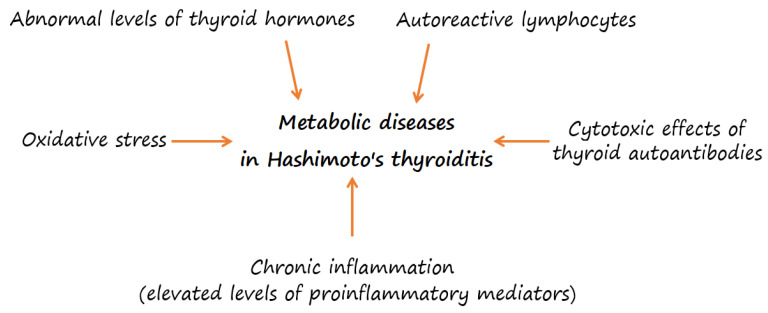
Factors influencing the increased risk of metabolic disorders in Hashimoto’s thyroiditis.

Obesity has become a major health problem worldwide in the past decade. It is caused by the excessive intake of food, low physical activity, and several other environmental factors that interact with genetic predisposition [34,35]. According to the latest data from the World Health Organization (WHO), in 2016, 39% of adults aged 18 years and older were overweight and 13% were obese [35]. In recent years, the incidence of metabolic and autoimmune thyroid diseases increased simultaneously, but there are limited studies that assessed the relationship of thyroid autoimmunity with the components of metabolic syndrome (MetS) and the risk of cardiovascular disease (CVD) in the presence of overt thyroid dysfunction. Some studies suggest that HT is associated with higher cardiovascular risk due to the autoimmune process, independent of thyroid function. However, the role of thyroid autoimmunity in promoting CVD remains inconsistent [36,37,38,39]. The increased risk of developing CVD in HT patients may be caused by proinflammatory mediators, the cytotoxic effects of autoantibodies, autoreactive lymphocytes and abnormal hormone levels (Figure 1) [40,41,42].

According to previous reports, obese people may be more prone to developing HT [43,44,45]. The latest systematic review and meta-analysis, including 22 studies, showed that obesity is significantly associated with HT and high levels of TPOAb [45]. Furthermore, a cohort study with 1277 women and 1185 men showed that childhood weight gain and overweight increased the susceptibility to later hypothyroidism and thyroid autoimmunity, especially in women [43]. Liu et al. in the study with non-obese euthyroid individuals, including 1402 AIT patients and 4206 sex-, age-, and body mass index (BMI)-matched healthy controls, showed that TPOAb levels was positively associated with total cholesterol (TC), triglycerides (TG), and homeostatic model assessment for insulin resistance (HOMA-IR) and high-sensitivity C-reactive protein (hsCRP) concentrations. Moreover, TPOAb level was an independent factor influenced hsCRP and HOMA-IR levels, suggesting that chronic inflammation and insulin resistance may be the link between thyroid autoimmunity and metabolic abnormalities in the non-obese cohort [46]. Another study with 50 patients diagnosed with subclinical hypothyroidism due to HT and 50 healthy controls showed that homocysteine (Hcy), hsCRP, and low-density lipoprotein (LDL) cholesterol levels were higher and high-density lipoprotein (HDL) cholesterol levels were lower in the study group compared with the control group [47]. Tamer et al. demonstrated that TC, LDL cholesterol, TG and non-high density lipoprotein (non-HDL) cholesterol levels, BMI and waist circumference (WC) of premenopausal women with HT were significantly higher compared to healthy subjects. This concerned not only overt hypothyroid HT patients but also subclinical and euthyroid HT patients. Moreover, TPOAb concentrations correlated positively with TG levels and negatively with HDL cholesterol levels and TgAb concentrations with TG and non-HDL cholesterol levels in all the patients with HT. This study showed that thyroid autoimmunity may have some effects on hyperlipidaemia and abdominal obesity independent of thyroid function, and is indicative of the need for determinations of lipid profiles and anthropometric parameters of all patients with HT, even euthyroid [39]. Another study confirmed that the euthyroid patients with HT have significantly higher body weight, BMI, waist-to-hip ratio (WHR) and fat mass than healthy individuals. In the HT group, 72% of the patients were overweight or obese, compared with 38% in the control group. Moreover, body weight, BMI and fat mass were higher in the patients treated with LT4 for less than two years compared to those treated for a longer period [48]. The cross-sectional study with 55,891 euthyroid subjects from China showed that in males, the BMI, WC, systolic blood pressure (SBP), diastolic blood pressure (DBP), and glucose level in a glucose tolerance test were significantly higher in the TPOAb-/TgAb-positive group than in the TPOAb/TgAb-negative group. In females, the BMI, WC, SBP, DBP, TC, and LDL cholesterol in the TPOAb/TgAb-positive group were significantly increased compared to controls. This study indicated that metabolic disorders are associated with increased positive thyroid autoantibody levels [49].

Lei et al. showed that in HT patients the serum thyroid hormone levels are closely related to blood lipid metabolism and inflammatory factors. The study with 91 patients with HT (42 with hypothyroidism and 49 euthyroidism) and 50 healthy people found that the levels of inflammatory factors, including interleukin 6 (IL-6), 12, 10 and tumor necrosis factor-α (TNF-α) were lower in the control group compared to the HT group. The level of HOMA-IR gradually declined in HT hypothyroid group, HT euthyroid group and healthy control group, adequately [50]. Moreover, hypothyroidism in the course of HT may also cause disturbances in carbohydrate metabolism. In the study conducted with 54 patients with HT, diabetes occurred in 27.8% of the patients. Impaired fasting glucose (IFG) or impaired glucose tolerance (IGT) were confirmed in 16.6% of HT patients, whereas a normal fasting glucose level was confirmed in 55.6%. Impaired carbohydrate metabolism, as well as with increased insulin resistance, occurred in almost half of the patients with HT [51]. Siemińska et al. conducted a study with subclinical hypothyroid and euthyroid postmenopausal women. The study showed that women with antithyroid antibodies exhibit higher serum IL-6 levels when compared to women without antibodies. An elevated level of this cytokine is a marker of endothelial dysfunction, which leads to the development of atherosclerosis. Moreover, HT subjects were more obese and had higher WC, WHR, SBP, and HOMA-IR compared to subclinical hypothyroid women without thyroid autoimmunity. Furthermore, the prevalence of MetS was higher in obese subclinical hypothyroid women with HT compared with those without thyroid autoimmunity. However, euthyroid women showed no difference in the prevalence of MetS between TPOAb-positive and negative groups [36].

Cerit et al. evaluated overt hypothyroid patients due to HT before and after six months of LT4 therapy when euthyroidism was achieved and compared to a healthy control group. A significant decrease in the body weight and BMI was observed after achieving euthyroidism compared to pretreatment values. In this study, the epicardial adipose tissue thickness (EATT) was higher in patients with hypothyroidism than in the control group, and decreased after six months of LT4 therapy but remained higher than in the control group, which may contribute to the development of atherosclerosis. The study also demonstrated that hsCRP levels were higher in the hypothyroid group, both before and after treatment than in the control group. Moreover, a significant reduction in Hcy and HOMA-IR levels was observed after the achievement of euthyroidism compared to the pretreatment values [52]. Others suggest that thyroid autoimmunity may lead to endothelial dysfunction, thus carotid intima-media thickness (CIMT) may be a helpful tool for the detection and monitoring of early atherosclerosis in euthyroid patients with HT. A study of newly diagnosed euthyroid pubertal girls with HT and healthy subjects demonstrated that there were no significant differences in anthropometric parameters, but patients with HT had significantly higher WHR. Thyroid hormones, Hcy, insulin, and HOMA-OR were similar. However, hsCRP, TC and LDL cholesterol levels were significantly higher in HT patients than in controls. The former also had significantly increased CIMT, a biomarker of subclinical atherosclerosis, regardless of thyroid function compared with controls [53]. Other studies confirmed higher CIMT levels in HT patients than in healthy controls and found that HT is an independent cardiovascular risk factor [54,55]. Topaloglu et al. also showed a positive correlation between TPOAb, TgAb levels and TC, LDL cholesterol and negative with HDL cholesterol. Therefore, more attention should be paid to patients with HT in terms of possible CVD, even if they are euthyroid [55].

The retrospective cohort analysis consisted of 1165 newly diagnosed HT patients and 4660 matched non-HT patients showed that the risk of developing coronary heart disease (CHD) in HT patients was increased. In this study, HT remained an independent risk factor for CHD after adjusting for comorbidities, including hypertension, hyperlipidemia, diabetes mellitus, stroke, chronic kidney disease, and heart failure. Moreover, the risk of CHD in HT patients decreased after treatment with LT4 for more than 1 year and did not differ from the non-HT group. HT patients without treatment and with treatment for less than 1 year were associated with a higher risk of CHD [42]. Collet et al. found no association between the presence of thyroid antibodies and CHD. They analyzed data from six prospective cohorts with 38,274 individuals and showed that the risk of CHD associated with subclinical hypothyroidism did not depend on TPOAb status, suggesting that thyroid autoimmunity biomarkers do not add independent prognostic information for CHD outcomes [56].

On the other hand, others have shown that euthyroid HT patients have similar metabolic and anthropometric measures and prevalence of metabolic syndrome compared to people without autoimmunity. Mousa et al. conducted a cross-sectional study with 301 euthyroid volunteers (99 with HT and 202 controls). The anthropometric and metabolic parameters, including BMI, fat mass, fasting insulin levels, HOMA-IR or TG were similar in both groups. The prevalence of MetS was also comparable in HT and control groups [57]. The study conducted in China with 1105 participants, mostly females, showed no differences in the mean BMI, the prevalence of diabetes, hypertriglyceridemia and hypercholesterolemia between participants with positive and negative thyroid autoantibodies [58]. Owecki et al. conducted a case-control study with 31 euthyroid females treated with LT4 due to HT, 26 women without LT4 therapy and 49 healthy females negative for TPOAb, all with comparable BMIs. Hcy levels were significantly decreased in treated HT patients as compared with healthy controls. Furthermore, there was no significant difference in Hcy levels between non-treated HT and the control group. The conclusions of this study stated that untreated euthyroid HT was not associated with an increase in Hcy. However, in this study it was shown that TPOAb levels were negatively correlated with HDL cholesterol and positively with WC [59]. The study by Solini et al. demonstrated no significant differences between newly diagnosed HT patients with BMI < 30 kg/m^2^ before therapy and healthy subjects in terms of lipid profile, except for serum TG, however, within the reference range. Euthyroid HT subjects with normal fasting glucose concentrations were characterized by increased levels of adipocytokines related to insulin sensitivity. However, the levels of fasting insulin and the HOMA-IR index were within the reference range and not significantly different from controls [60].

Some studies suggested that patients with HT, even euthyroid, have significantly worse anthropometric parameters and metabolic outcomes than healthy individuals. On the other hand, others pointed out that, although obesity or overweight is frequently encountered in patients with HT, they have similar metabolic and anthropometric parameters compared to healthy subjects. Therefore, other factors may also influence the metabolic parameters in HT.

## 4. Oxidative Stress in HT

Oxidative stress is a result of the overproduction of free oxygen radicals and impaired antioxidant defense [9,61,62]. The most important prooxidants are the reactive oxygen species and reactive nitrogen species [61]. The balance between prooxidants and antioxidants is essential for the proper functioning of the thyroid gland. Recent evidence has confirmed altered antioxidant potential and increased oxidative stress in HT patients. Many studies have shown that oxidants are increased and antioxidants decreased in patients with HT, irrespective of the thyroid functional status (both in euthyroidism and overt hypothyroidism) compared to healthy subjects. Therefore, the balance between oxidants and antioxidants is shifted toward the oxidative side [62,63,64,65,66,67].

The study by Ruggeri et al. showed that advanced glycation end products (AGEs) levels were higher in 71 newly diagnosed untreated euthyroid HT patients than in 63 healthy controls. Furthermore, serum derived reactive oxygen metabolites (d-ROMs) were increased and the biological antioxidant potential (BAP) test decreased in HT subjects compared with controls. Serum TPOAb was the predictor for d-ROMs, BAP, and AGEs, irrespective of TSH and free thyroxine (fT4) values [66]. The study with 44 newly diagnosed females with HT and 58 controls showed that HT patients exhibited markedly reduced mean serum glutathione (GSH) levels, a defense against oxidative damage. In HT patients, significant associations were seen between GSH and TPOAb, GSH and TSH, respectively. This study showed that GSH deficiency initiates oxidative stress and the development of immune intolerance in the course of HT [63]. Morawska et al. observed that total antioxidant potential (TAC) was significantly lower (by 82%), while total oxidant status (TOS), oxidative stress index (OSI) and the level of oxidation products of proteins (AGEs) and lipids (lipid hydroperoxides) were significantly higher in HT patients compared to the control group. Moreover, the saliva of euthyroid patients with HT also demonstrated a reduced antioxidant potential [68]. Korkmaz et al. showed that the activity of serum antioxidant enzyme paraoxonase-1 (PON1) was significantly lower in 25 euthyroid HT patients compared to 27 healthy subjects [69].

Recent studies indicated that high oxidative stress is associated with the severity and progression of HT from euthyroidism to overt hypothyroidism [65,70]. Ates et al. conducted a study with newly diagnosed HT patients (31 in each stage: euthyroid, subclinical and overt hypothyroid) without treatment and 31 healthy volunteers. The oxidative stress level was higher in all stages of HT when compared to the control group, and there was a negative correlation between total antioxidant balance and thyroid autoantibodies. Moreover, TOS and OSI levels were increased, and total antioxidant status (TAS) was decreased in overt hypothyroid patients compared to other groups. These results suggest that oxidative stress continues to increase during the exacerbation of hypothyroidism in HT patients [65]. A later study also conducted by Ates et al. showed that after 9 months of follow-up of euthyroid and subclinical hypothyroid HT patients without treatment, 17.5% of them developed overt hypothyroidism. TOS and OSI were higher in patients who developed overt hypothyroidism than in those who did not. However, no significant difference was found in PON1 levels between groups. Moreover, there was a positive correlation between oxidant parameters and thyroid autoantibodies. The authors concluded that oxidative stress may be a risk factor for overt hypothyroidism in HT patients [70]. Another study with 30 subclinical HT, 18 overt hypothyroidism HT patients and 30 healthy controls confirmed a significant increase in oxidative stress parameters and LDL cholesterol in HT patients, especially with overt hypothyroidism [67].

Other studies have shown that LT4 therapy in hypothyroid HT patients decreases oxidant and increases antioxidant status. After six months of LT4 therapy in the HT-related hypothyroidism group, serum TAS and PON1 levels increased and serum TOS and OSI levels decreased significantly. Furthermore, pretreatment serum TAS and PON1 levels were positively correlated with fT4 and negatively correlated with TSH, TPOAb and TgAb levels. Additionally, serum TOS and OSI levels were negatively associated with fT4 levels and positively correlated with TSH, TPOAb, and TgAb. It was also found that the fT4 and TPOAb concentrations are independent predictors of the oxidative stress parameters [71]. In another recent study of 218 euthyroid HT women treated with LT4 and without treatment, the high total lipid peroxide levels were found to be more frequent in women receiving LT4. Oxidative stress was increased in overweight and obese women with HT compared to the women with normal BMI. Moreover, low fruit and vegetable consumption were associated with increased “high TOS” prevalence. The results of this study indicate that a normal BMI and daily consumption of fruit and vegetables contribute to maintaining oxidative stress at a low level. Additionally, LT4 treatment, excessive BMI, low fruit and vegetable consumption were significant independent predictors of high oxidant stress in HT [62].

Oxidative stress may be a significant risk factor in the pathogenesis and progression of HT and the development of complications. Therefore, appropriate lifestyle changes should be implemented, including diet and body weight normalization to reduce oxidative stress in these patients. Some vitamins, such as vitamin A, E, or C, and minerals, including Se, which are found in fresh and raw foods, exhibit antioxidant properties. Frequent consumption of these foods reinforces antioxidant defense mechanisms [72].

## 5. Micronutrients in HT

### 5.1. Iodine

Iodine is a necessary microelement of the diet for the proper functioning of the thyroid gland, including the synthesis of triiodothyronine (T3) and thyroxine (T4). The primary strategy for the elimination of iodine deficiency is universal salt iodization, which began in the 20th century. The addition of iodine to the salt has solved the problem of iodine deficiency in many countries around the globe. Currently, due to the increased incidence of hypertension and other CVD, it is recommended to limit salt intake, which is the main source of iodine [73,74]. According to the WHO, the adequate daily iodine intake is 150 μg/day in adults [75]. The main sources of iodine in the diet are seafood, fishes, milk, dairy products, vegetables, and fruits [74,76].

Iodine is important not only for the synthesis of thyroid hormones but also affects the induction and modulation of thyroid autoimmunity. Research suggests that an excess of iodine stimulates thymus development and affects the functioning of various immune cells [77]. The role of excessive amounts of iodine in inducing thyroid autoimmunity has been demonstrated in animal models. The mechanisms by which iodine causes thyroiditis are still unclear. Several explanations have been proposed, including the induction of cytokine and chemokine production by excess iodine that can recruit immunocompetent cells to the thyroid gland. Other mechanisms include the processing of excess iodine in thyroid epithelial cells, which resulted in increased levels of oxidative stress, harmful lipid oxidation and caused damage to the thyroid tissue or increased the immunogenicity of highly iodized thyroglobulin [78]. Excessive iodine intake is associated with a higher incidence of AIT, while in areas with iodine deficiency, a lower prevalence has been demonstrated [2].

**Figure 2:**
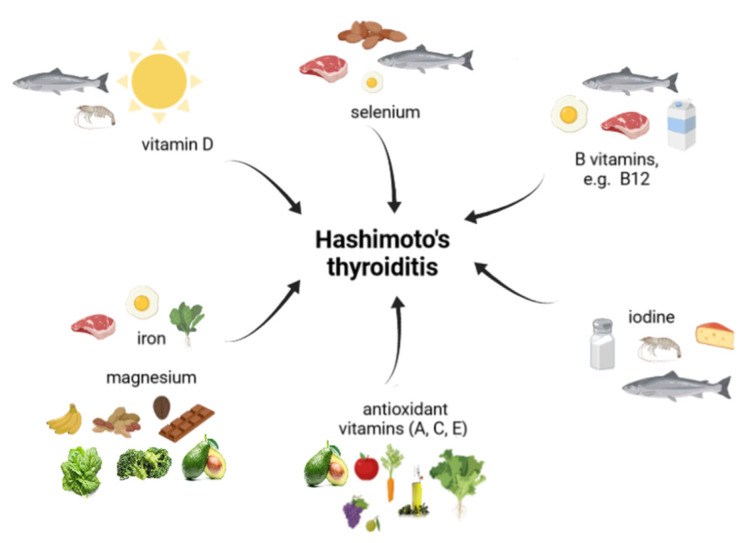
Important nutritional microelements in Hashimoto’s thyroiditis.

Excessive iodine intake or over-supplementation induced thyroid dysfunction, including thyroid autoimmunity, and therefore it is not recommended in HT patients. Monitoring the body iodine status allows for the avoiding of both iodine deficiency and excess [74,78,79]. Figure 2 presents the essential microelements of which an adequate supply is required in HT.

### 5.2. Selenium

Se is an essential micronutrient with many pleiotropic effects, including antioxidant and anti-inflammatory properties [80]. The thyroid gland has the highest Se content per gram of tissue, because it expresses specific selenoproteins [60,80]. Selenoproteins such as glutathione peroxidases and iodothyronine deiodinases play an important role in human thyroid function [81,82]. Therefore, Se supplementation, especially in the form of selenomethionine, may be beneficial in HT patients with Se deficiency and adequate iodine intake [74]. Selenium in foods is most often found in combination with proteins, therefore it can be present in products such as meat, marine origin and freshwater fish, eggs, seafoods, offal, and cereals. However, Brazil nuts and mushrooms have the highest selenium content. Other sources include dairy products, onion, garlic and plants of the Brassica genus (broccoli, cabbage and cauliflower) [83]. So far, none of the European or American Endocrine/Thyroid Societies recommended Se supplementation in HT [84]. Recent studies have shown that only 20% of European Thyroid Society (ETA) members claimed that the available evidence supports the prescription of Se supplements in patients with HT. A previously mentioned study also demonstrated that Se was advised by 69% of ETA members for HT patients not receiving LT4 and 50% with LT4 treatment. Supplementation was expected to reduce levels of circulating thyroid autoantibodies, slow down the increase of TSH concentration and improve the health-related quality of life or thyroid morphology [85].

Rostami et al. conducted a study with 49 newly diagnosed HT females and 50 controls. HT patients exhibited lower Se levels compared to controls. Moreover, 58.8% of HT patients and 34% of healthy controls had Se deficiency. Se-deficient patients had higher levels of TSH, TPOAb and TgAb compared to Se-sufficient patients. In this group, negative correlations were observed between Se concentration and TSH, and TPOAb levels. This study highlighted the importance of proper Se status in HT, indicating the need for further studies to evaluate the clinical benefits of improving antioxidant status in HT [28]. In a recent study by Kryczyk-Kozioł et al., 29 newly diagnosed and untreated HT women with euthyroidism or subclinical hypothyroidism received 100 µg of Se daily for six months. The results of this study demonstrated the protective effect of Se in limiting the development of overt hypothyroidism. Se supplementation significantly decreased the level of TPOAb. This may stabilize thyroid function, as thyroid parameters (TSH, free triiodothyronine (fT3), fT4), had similar values before and at the end of the study [86]. Another study also confirmed that in patients with HT, TgAb and TPOAb titers decreased after Se supplementation. In a recent study by Wang et al., 100 patients with HT, Se deficiency and euthyroidism received 200 µg of Se daily for six months. After three and six months of therapy, the overall serum Se level in patients increased significantly to a moderate level. Moreover, TPOAb and TgAb decreased after six months of Se supplementation compared with baseline [87]. Tian et al. confirmed that Se supplementation decreased TPOAb titer by enhancing defense against oxidative stress in euthyroid patients with AIT [30]

Some authors suggested that Se supplementation supports the treatment with LT4. A systematic review and meta-analysis by Wichman et al. demonstrated that Se supplementation reduced TPOAb levels after three, six, and 12 months in LT4-treated patients with HT, and after three months in an untreated population. TgAb decreased at three months, but not after six or 12 months. However, more studies are needed to show whether these observations are clinically relevant [31].

Only a few studies have reported changes in TSH levels following Se supplementation. The prospective study assessed the effects of Se supplementation on TSH levels in 45 patients with subclinical hypothyroidism due to HT. They received 83 µg of selenomethionine/day for four months without LT4 treatment. After this time, euthyroidism was restored in 48.9% of participants (study group), while the rest of the patients remained hypothyroid (control group). There were no significant changes in TPOAb levels from baseline to the end of the study in both groups. Moreover, six months after selenomethionine withdrawal, 83.3% of the study group remained euthyroid, while only 14.2% of the control group became euthyroid. This study showed that Se supplementation is associated with the normalization of serum TSH levels [88]. The recent prospective, randomized controlled study evaluated the clinical effect of Se supplementation in patients with HT without T4 replacement and suggested the potential mechanism against thyroid autoimmunity. The study group (n = 43) received a Se yeast tablet for six months, while the control group was not treated (n = 47). The results showed that Se supplementation significantly decreased TPOAb, TgAb, and TSH levels, accompanied by increased selenoproteins, such as glutathione peroxidase 3 and selenoprotein P1, compared with the control group [89].

In 2019, Karimi and Omrani conducted a surprising clinical trial with 102 patients with HT. After 3 months of daily 200 μg Se supplementation (group 1), as well as 500 mg of vitamin C (group 2), both with antioxidant properties, a significant reduction in TPOAb levels occurred. In this regard, there was no significant difference between the two groups. Moreover, in group 3, which received a placebo, TPOAb concentrations did not change. In this study, there was no significant difference in TSH and TgAb levels between all three groups. These findings confirmed the antioxidant beneficial effects of Se in HT patients. However, Se supplementation was just as effective as vitamin C regarding its effects on thyroid-specific antibodies [90].

Some studies did not show a beneficial effect of Se supplementation in HT patients [91,92]. A blinded placebo-controlled randomized prospective study with newly diagnosed euthyroid HT patients described a limited effect of short-term Se supplementation. In this study, 38 HT patients received a placebo, and the other 38 received l-selenomethionine 166 μg/day for six months. TSH, fT4, fT3 and TPOAb concentrations were not different between the study and control groups at a baseline, after three and six months [91]. Moreover, the systematic review by van Zuuren et al. concluded that the clinical relevance of Se supplementation in patients with HT is unclear. The studies demonstrated insufficient evidence to support the efficacy of Se in patients with HT [92]. Further studies are needed to prove that Se supplementation in patients with HT may prevent the progression to hypothyroidism.

### 5.3. Iron and Magnesium

In patients with HT, apart from iodine and Se, it is necessary to ensure an adequate supply of iron and magnesium. Thyroid peroxidase is a haeme enzyme responsible for the production of thyroid hormones which becomes active after binding haem. Therefore, iron deficiency may impair thyroid metabolism. Iron in foods occurs in two forms: haeme and non-haeme. Red meat is rich in hemoglobin and is therefore the main source of iron. In addition, haeme iron is found in poultry, eggs and fish, while non-haeme iron is obtained from cereals, legumes, vegetables and fruits [93]. The association between iron deficiency and thyroid autoimmunity has not been well established. Many patients with HT are iron deficient due to comorbidities such as autoimmune gastritis, which causes a reduction in iron absorption, or celiac disease, which leads to iron loss [81,82,94]. Some studies have shown that iron treatment in women with anemia and impaired thyroid function improves thyroid hormone levels [81,82]. In a recent retrospective study, the levels of TSH, TPOAb and TgAb were significantly higher, while hemoglobin, hematocrit, MCV, ferritin and iron were significantly lower in 180 female patients with positive thyroid autoantibodies compared to 81 healthy controls. In patients with thyroid autoantibodies, the frequency of abnormal levels of hemoglobin, iron, and ferritin was higher than in controls. A negative correlation was found between TPOAb and serum ferritin and iron levels in the patient group. This study confirmed that patients with AIT had a higher risk of developing iron deficiency [95]. Moreover, a cross-sectional analysis of 7463 pregnant women and 2185 non-pregnant women with subclinical hypothyroid showed that the prevalence of isolated TPOAb-positivity was significantly higher in women with iron deficiency than in those without iron deficiency for both groups [96]. A recent systematic review and meta-analysis summarized the effects of iron deficiency on thyroid function and autoimmunity. Iron deficiency significantly increases the risk of positive TPOAb and both positive TPOAb and TgAb in women of reproductive age. This study indicates that it is necessary to monitor the nutritional status of iron in AIT [97].

Another relevant trace element is magnesium. It is one of the most abundant elements in the human body as a cofactor for over 300 enzymes that regulate a variety of biochemical processes. Magnesium is a mineral found in plant and animal foods and beverages. Good sources of magnesium include greens, like spinach, broccoli and avocado, legumes, nuts, almonds, seeds, bananas, and whole grains [98]. Decreased serum levels of magnesium are associated with several chronic diseases, however, its association with HT is unclear. The cross-sectional study with 1257 participants showed that low serum magnesium levels were associated with increased risk of TgAb positivity, the prevalence of HT, and hypothyroidism. However, no relationship was found between TPOAb and magnesium levels. The results of this study indicated that magnesium levels are worth assessing in patients with HT and hypothyroidism. Accordingly, more research is needed to elucidate the association between serum magnesium levels, AIT and thyroid function [99].

### 5.4. Vitamin D

Recent evidence points to various roles of vitamin D, including cell proliferation and differentiation and immunomodulation, therefore, its supplementation seems to be effective in reducing the level of anti-thyroid antibodies in HT patients [100]. Vitamin D is mainly produced endogenously after exposure to sunlight on the skin (specifically UVB radiation). Food consumption is a minor source of this vitamin. Among the products that contain the highest amounts of vitamin D are fatty fish (such as salmon and mackerel) and fish liver oils. Other sources of this vitamin include meat, offal, egg and dairy products [101]. The use of vitamin D supplements has increased significantly in recent years. The role of vitamin D supplementation and the optimal dose is the subject of many studies. Vitamin D deficiency has been a worldwide problem over the years. It is defined by a serum 25-hydroxyvitamin D [25(OH)D] level below 50 nmol/L or 20 ng/mL [102]. In recent years, many studies searched for the association between HT and vitamin D deficiency [103,104,105,106,107].

The role of vitamin D in the pathogenesis of HT is unclear, although some studies have found an association between vitamin D deficiency and the presence of thyroid autoantibodies. The epidemiological studies from 2021 showed that vitamin D deficiency was associated with thyroid autoantibody positivity. Moreover, HT patients had significantly higher concentrations of cytokines secreted by the proinflammatory Th1 and Th17 cells, i.e., interferon-gamma (IFN-γ) and IL-17, compared to the healthy subjects. The study showed that sufficient vitamin D concentration contributes to adequate immune tolerance by regulating the differentiation of CD4+ T-cells. Therefore, vitamin D deficiency seems to be involved in the pathological mechanism underlying HT and more randomized controlled studies are needed to show whether vitamin D supplementation can be used to treat HT or delay its progression [104].

A cross-sectional study by Mansournia et al. with 41 HT patients with overt or subclinical hypothyroidism and 45 healthy euthyroid controls suggested that higher serum vitamin D levels were associated with decreased risk of HT after adjustment for potential confounding factors, including age, sex, and BMI. However, there was no significant correlation between serum 25(OH)D and TPOAb levels [108]. In a cohort study of 261 healthy overweight and obese subjects, low 25-hydroxyvitamin D levels have been associated independently with HT. The analysis showed that vitamin D deficiency was significantly higher among the population with HT than in those without HT [109]. One other study confirmed that vitamin D level is an independent factor affecting the presence of TPOAb in AIT [110]. The study in the Polish-Caucasian population showed that serum levels of 25(OH)D were significantly lower in HT patients compared to the healthy control group. Therefore, the authors also suggested that vitamin D deficiency is one of the risk factors for HT development, although changes in vitamin D levels may occur as a result of the disease [111].

In a cross-sectional study, the prevalence of vitamin D insufficiency (<30 ng/mL) was significantly higher in the patients with HT than in non-AIT. Among the HT population, patients with overt hypothyroidism had lower 25(OH)D levels and a significantly higher prevalence of vitamin D insufficiency compared with those with euthyroidism and subclinical hypothyroidism or those without AIT. Moreover, low serum vitamin D status was independently associated with high TSH levels [112]. Another study confirmed that serum vitamin D levels are lower in women with AIT and primary hypothyroidism compared to euthyroidism. This study also revealed a negative association between TSH and vitamin D levels, antithyroid peroxidase antibodies and vitamin D levels in women, but not in men [113].

Chao et al. studied 5230 individuals and found that the level of 25(OH)D in the group without HT was higher than in the HT group. Besides, the presence of HT was significantly positively correlated with BMI, WC and TSH. Moreover, fT3 and fT4 levels were positively correlated with vitamin D, but there was no significant correlation between 25(OH)D and TPOAb or TgAb. This study indicated that HT patients have decreased vitamin D levels and that TSH is an independent risk factor for HT [107]. Interestingly, low vitamin D levels were associated with cognitive impairment in patients with HT. The study showed that 28.4% of HT patients were diagnosed with mild cognitive impairment (MCI). Moreover, serum 25(OH)D levels were significantly associated with MCI in patients with HT [114].

Few recent systematic reviews and meta-analyses confirmed significantly lower vitamin D levels in patients with HT compared to healthy subjects [103,105,106].

On the contrary, some studies showed no differences in vitamin D levels in patients with HT and the healthy subjects. Two case-control studies by Effraimidis et al. showed no associations between vitamin D deficiency and a higher prevalence of HT [115]. The study with 88 patients with HT and 71 euthyroid healthy subjects showed that vitamin D concentrations were similar in both groups. However, in HT patients there was a positive correlation between vitamin D and fT4, IL-17 and TNF-α. Moreover, the reduction of fT4 levels was a predictor of vitamin D insufficiency for HT patients, but not for the control group [116]. Another cross-sectional and retrospective study with 461 HT patients and 176 controls also showed no significant differences in vitamin D levels between HT patients and control participants. However, the results of this study showed decreasing vitamin D levels along with disease progression. There was only a nominally negative correlation between vitamin D and TSH in HT patients. Therefore, it is worth considering vitamin D supplementation, especially for HT patients with the more severe form of the disease [117].

Recently, after the analysis of vitamin D concentrations, the consequences of vitamin D supplementation in HT patients were investigated. Table 2 shows the effects of vitamin D supplementation on circulating thyroid autoantibodies and the thyroid profile in HT patients. Studies on the mechanisms of vitamin D action and the pathogenesis of HT have shown that vitamin D can reverse thyroid cell damage caused by autoreactive immune cells. The clinical trial study with 48 female patients with HT showed that weekly supplementation of 50,000 IU vitamin D for 3 months caused a significant decrease in the Th17/Tr1 ratio. These results have shown that vitamin D supplementation induces beneficial effects on T-cell homoeostasis disorders in HT patients. However, more research is needed to confirm the effects of vitamin D treatment on immune function in patients with HT [118].

Ucan et al. found a positive effect of vitamin D supplementation on patients with HT. Vitamin D administration determined a decrease in thyroid autoantibody levels. Moreover, HDL cholesterol concentrations increased in the euthyroid HT group after treatment. Therefore, vitamin D supplementation may slow the development of hypothyroidism and reduce cardiovascular risk [119].

Mazokopakis et al. studied euthyroid patients with HT who lived on the sunny island. At the beginning of the study, TPOAb levels were significantly higher in vitamin D deficient HT patients compared to vitamin D sufficient patients. The vitamin D deficient HT patients received vitamin D supplementation, therefore serum 25(OH)D levels increased significantly and TPOAb levels decreased. These findings indicated that vitamin D deficiency may be related to the pathogenesis of HT and that its supplementation could support the treatment of patients with HT [27]. The prospective study by Simsek et al. showed that TPOAb and TgAb levels were significantly decreased after vitamin D supplementation in AIT patients with vitamin D levels < 20 ng/mL, whereas the evaluated parameters did not change substantially in patients without therapy [120]. An open-labelled randomized controlled trial with newly diagnosed AIT patients demonstrated that 93% had vitamin D insufficiency (25(OH)D < 30 ng/mL). After three months of vitamin D and calcium supplementation, there was a significant fall in TPOAb in the study group compared to the control group, which received only calcium [121].

A systematic review and meta-analysis from 2021 which included 8 studies (n = 652) also suggests that vitamin D supplementation for >three months reduces autoantibody titers in patients with HT, although treatment ≤3 months was ineffective [32]. Another recent systematic review and meta-analysis which included six randomized controlled trials with 330 HT patients also showed that after six months of vitamin D supplementation, TPOAb and TgAb levels significantly decreased as compared with the control group. This analysis confirmed a positive effect of vitamin D supplementation in HT. However, more long-term studies are needed to further confirm the supplementation effectiveness [33].

The retrospective study with euthyroid subjects with and without AIT, all diagnosed with vitamin D deficiency, taking supplements with 25(OH)D for various periods of time and different doses, searched for TSH changes. This study showed that in patients with AIT, TSH levels significantly decreased after 100,000 IU of vitamin D therapy monthly, whereas no significant variation of TSH was observed in the control group, irrespective of treatment dose and duration. The results of this study suggest that treatment with vitamin D may improve thyroid function [122]. Another study demonstrated that vitamin D administration decreased thyroid antibody titers, but did not affect serum levels of TSH and free thyroid hormones in HT women with serum 25(OH)D levels above 30 ng/mL, treated with LT4. Moreover, this study showed that HT patients with hypothyroidism may benefit more from vitamin D therapy than euthyroid patients. However, larger prospective trials are needed to support this observation [123].

Some studies suggest that taking Se may enhance the effects of vitamin D. A comparative study involving 47 euthyroid women with HT who supplemented 4000 IU of vitamin D daily, and 23 of them who additionally took selenomethionine (200 μg daily), showed that 25-hydroxyvitamin D levels increased in both groups and reduced TPOAb and TgAb titers. In addition, the effect on antibody titers was more pronounced in the group receiving selenomethionine [124].

In opposition to the aforementioned, no effect of vitamin D supplementation in HT patients was shown. Some studies have found no significant reduction in TPOAb levels in HT patients after vitamin D supplementation compared to the placebo group. A double-blind, randomized clinical trial with HT women showed a significant reduction of TgAb and TSH levels in the vitamin D supplement group compared to the start of the study [125]. Another double-blind randomized placebo-controlled clinical trial showed that vitamin D supplementation in patients with HT and vitamin D deficiency could not have a significant effect on thyroid function and autoimmunity. This study demonstrated that vitamin D levels were increased after 12 weeks in the supplemented group. However, no significant differences were observed between groups with regard to TPOAb and TSH levels [126].

Most studies demonstrated that vitamin D supplementation significantly increases serum 25(OH)D levels and induces changes in TPOAb levels in patients with HT. However, in the majority of cases, no significant associations were found between serum vitamin D and TgAb, TSH, fT3, and fT4 concentrations.

**Table 2:** Effect of vitamin D supplementation on selected parameters in patients with Hashimoto’s thyroiditis—recent clinical studies.

Study	Study Population	Dose/Form/Supplementation Time	Evaluated Diagnostic Parameters	Key Results	Ref.
**Clinical trial**	218 euthyroid HT patients	186 HT patients with 25(OH)D < 30 ng/mL received 1200–4000 IU vitamin D daily for four months	Anthropometric characteristics, SBP, DBP, serum concentrations of vitamin D, TSH, fT4, calcium, phosphorus, titers of TPOAb and TgAb	- significant negative correlation between 25(OH)D and TPOAb levels among all HT patients; - TPOAb levels were significantly higher in vitamin D deficient HT patients compared to no vitamin D deficiency; - supplementation of vitamin D in vitamin D deficient HT patients caused a significant decrease (20.3%) in serum TPOAb levels; - after supplementation BMI, serum TgAb and TSH levels decreased not significant.	[27]
	75 patients with HT and 43 healthy individuals	Vitamin D deficient patients (<20 ng/mL): 50,000 IU of 25(OH)D3 weekly for eight weeks	Serum levels of vitamin D, TSH, HDL cholesterol and thyroid autoantibodies titers	- patients with HT had significantly lower vitamin D level compared to controls; - titers of thyroid autoantibodies significantly decreased after vitamin D supplementation in euthyroid HT patients; - after supplementation HDL cholesterol level improved in the euthyroid HT group.	[119]
**Prospective study**	82 patients with AIT and vitamin D deficiency (<20 ng/mL)	*Study group* (n = 46): patients treated with 1000 IU/day vitamin D for one month; *Control group* (n = 36): patients without vitamin D therapy	Serum levels of vitamin D, TSH, fT4, titers of TPOAb and TgAb	- TPOAb and TgAb levels were significantly decreased in the study group, this evaluated parameters did not significantly change in the control group; - thyroid function tests did not significantly change in two groups.	[120]
**Randomized controlled trial**	100 newly diagnosed AIT patients	*Study group* (n = 50): 60,000 IU 25(OH)D weekly and 500 mg/day calcium for eight weeks; *Control group* (n = 50): 500 mg/day calcium for eight weeks	Serum levels of vitamin D and titers of TPOAb	- 74% of HT patients were vitamin D deficient; - significant decrease of TPOAb level in the study group compared to the control group.	[121]
**Retrospective study**	198 euthyroid subjects with vitamin D deficiency (<30 ng/mL)	*Study group* (n = 95): patients with AIT; *Control group* (n = 103): subjects without AIT. The groups were also divided into subgroups depending on the dose and period of supplementation	Serum levels of vitamin D and TSH	- in the study group TSH level significantly decreased after 100,000 IU vitamin D monthly; - no significant changes in TSH level in the control group, regardless of treatment dose and duration; - 25(OH)D level significantly improved with all monthly doses and especially in the group receiving 100,000 IU/month.	[122]
**Clinical Trial**	34 euthyroid or mild subclinical hypothyroid HT women with 25(OH)D levels >30 ng/mL, treated ≥6 months with LT4	*Study group* (n = 18): women with LT4 and 2000 IU vitamin D daily for six months; *Control group* (n = 16): women with LT4 treatment	Serum levels of vitamin D, TSH, fT4, fT3, titers of TPOAb and TgAb	- in the study group 25(OH)D levels increased, TPOAb titers reduced and tended to reduce TgAb; - vitamin D supplementation did not affect serum levels of TSH, fT3 and fT4; - 25(OH)D level inversely correlated with titers of thyroid antibodies.	[123]
**Randomized clinical trial**	42 women with HT	*Study group:* 50,000 IU vitamin D weekly for three months; *Control group:* placebo for three months	Serum levels of vitamin D, Ca^2+^ ion, T4, T3, TSH titers of TPOAb and TgAb	- significant decrease of TgAb and TSH levels in the study group; - no significant reduction of TPOAb level in the study group compared to controls; - no significant changes in the serum levels of T3 and T4 levels in both groups.	[125]
**Double-blind, randomized, placebo-controlled clinical trial**	56 patients with HT and vitamin D deficiency (≤20 ng/mL)	*Study group* (n = 30): 50,000 IU vitamin D weekly for 12 weeks; *Control group* (n = 26): placebo for 12 weeks	Serum levels of vitamin D, TSH, calcium, parathormone, creatinine and TPOAb titers	- vitamin D level increased in the study group; - TPOAb and TSH levels did not significantly change in both groups; - significant decrease for PTH level in study group.	[126]
**Double-blind, randomized, placebo-controlled clinical trial**	65 vitamin D deficient euthyroid or hypothyroid patients with positive TPOAb	*Study group* (n = 33): 50,000 IU vitamin D3 weekly for 12 weeks; *Control group* (n = 32): placebo for 12 weeks	Serum levels of calcium, hsCRP, insulin, albumin, phosphorus, TG, TC and HDL cholesterol, IFG, glycated hemoglobin (HbA1c), blood urea nitrogen, creatinine	- levels of vitamin D increased significantly in study group; - HbA1c and insulin levels was increased significantly in both groups; - other variables did not change a significantly after trial.	[127]
**A Randomized Open-label Trial**	23 patients with HT	Weekly supplementation of 60,000 IU vitamin D for eight weeks followed by once a month for four months	Serum levels of vitamin D, TSH, fT4, and TPOAb titers	- serum vitamin D level was increased significantly after trial (87% patients had normal levels); - significant increase in the TPOAb and fT4 levels and significant reduction of TSH level after six months of therapy.	[128]
**Comparative Study**	47 euthyroid women with HT and low vitamin D status	*Study group* (n = 23): 200 μg selenomethionine daily for at least 12 months before the study and 4000 IU vitamin D daily for six months *Control group* (n = 24): 4000 IU vitamin D daily for six months	Serum levels of TSH, fT4, fT3, vitamin D, titers of TPOAb and TgAb	- in both groups, 25(OH)D levels were increased, TPOAb and TgAb titers were reduced; - the effects on antibody titers were more pronounced in women receiving vitamin D and selenomethionine;	[129]

HT—Hashimoto’s thyroiditis; n—Number of participants; LT4—Levothyroxine; TPOAb—Anti-thyroid peroxidase antibodies; TgAb—Anti-thyroglobulin antibodies; TSH–Thyrotropin; fT4—Free thyroxine; fT3—Free triiodothyronine; 25(OH)D—25-hydroxyvitamin D; IU—International unit; hsCRP, TC—Total cholesterol; TG—Triglycerides; HDL—High-density lipoprotein; IFG—Impaired fasting glycaemia; HbA1c—Glycated hemoglobin; SBP—Systolic blood pressure; DBP—Diastolic blood pressure.

In recent years, increasing evidence has also been documented that some vitamin D receptor polymorphisms, including rs2228570 (FokI), rs1544410 (BsmI), rs7975232 (ApaI), and rs731236 (TaqI) could be related to an increased incidence of HT [130]. However, not all studies support these findings [131].

### 5.5. Vitamin B12

As mentioned earlier, in patients with HT, the risk of anaemia may be increased by concomitant autoimmune diseases such as pernicious anaemia or atrophic gastritis. Pernicious anaemia is a type of anaemia caused by vitamin B12 deficiency that frequently coexists with HT and occurs at any age [132,133,134]. Vitamin B12 is present in foods of animal origin, including meat, fish, eggs, milk, and other dairy products [135]. The study with 130 patients diagnosed with autoimmune hypothyroidism found that vitamin B12 deficiency was found in 46% of patients and was associated with the presence of this disease. Moreover, the TPOAb levels were significantly higher in patients with low vitamin B12 levels and there was a negative correlation between vitamin B12 and TPOAb antibodies. Therefore, the researchers indicated that in these patients, the concentration of vitamin B12, as well as vitamin D, should be tested at the time of diagnosis and periodically as a part of follow-up testing. In addition, vitamin B12 deficiency may lead to increased Hcy levels, which, in consequence, may cause the development of atherosclerosis [134]. Kumari et al. demonstrated with 350 patients with AIT that the mean vitamin B12 level was 204.6 pg/mL. Moreover, 45.5% had serum vitamin B12 values below the lower limit of the reference range (<178 pg/mL) and 55% below 200 pg/mL, although there was no significant correlation between B12 and TPOAb [136]. The study with 190 TgAb- or TPOAb-positive patients, mostly euthyroid and 190 age- and sex-matched healthy control subjects, showed that patients had a significantly higher frequency of hemoglobin, iron or vitamin B12 deficiency and elevated Hcy levels than controls [137]. More research is needed to analyze the risk of vitamin B12 deficiency in HT patients.

An appropriate amount of trace elements such as iodine, Se, iron, magnesium and vitamins, especially vitamins D and B12, is essential for the health of the thyroid gland.

## 6. Diet in HT

Eating habits may affect the risk of several inflammatory and immune diseases, including autoimmune diseases. The current knowledge of the diet in HT is insufficient. There is a lack of comprehensive studies investigating dietary habits and their influence on HT. Only a few studies have analyzed the prevalence of HT concerning dietary habits [62,138,139,140,141]. Moreover, there are still no nutritional guidelines dedicated to HT patients. They rely only on the individual expert’s opinions and advice appearing in the media, which provide incomplete and often contradictory information [142,143].

### 6.1. Different Eating Patterns in HT

A study conducted by Ihnatowicz et al. consisting of 406 patients with HT showed internally differentiated eating patterns, and labelled them as ‘Convenient’, ‘Non-meat’, ‘Pro-healthy’ and ‘Carnivores’. Their various food product choices, experiences with diets, nutritional knowledge as well as food allergies and intolerances, indicated a large variability of diet in the population [142].

Some recent studies found a positive association between animal fat consumption and increased TPOAb and/or TgAb [139,140]. Saturated fatty acids contained in animal fats can induce an inflammatory response, including the expression of proinflammatory transcription factors that induce inflammatory mediators (including cytokines), which affects the development and progression of many chronic diseases [144]. Recent studies on a rat model have shown that a high-fat diet significantly increases TSH levels and causes thyroid dysfunction, suggesting that excessive consumption of animal fat induces thyroid dysfunction and may contribute to the pathogenesis of hypothyroidism [145,146]. A study by Matana et al. with 1887 participants, including 462 people with elevated TPOAb and/or TgAb levels, analyzed the relationship between dietary factors and the level of thyroid antibodies. They showed that high consumption of animal fats and butter was associated with positive TPOAb and/or TgAb, while frequent consumption of different sorts of vegetables, dried fruit, legumes, nuts, and muesli were negatively associated with the TPOAb and/or TgAb. This study suggests that the anti-inflammatory diet, based on products rich in polyphenols and phytosterols, is associated with negative TPOAb and/or TgAb levels [139]. This is one of the largest studies on the diet of people with HT to date. Another study with 491 patients with HT and 433 controls found significantly increased consumption of animal fat and processed meat in the HT group, whereas healthy individuals more frequently consumed whole grains, plant oil, olive oil, oily fish, fruits, red meat and non-alcoholic beverages. In addition, the study showed no differences in dietary habits in patients with HT with and without LT4 therapy except for red meat consumption, whereas patients on LT4 therapy consumed significantly more red meat. This finding suggests that HT patients do not modify their diet after diagnosis [140].

Ruggeri et al. conducted a study with 81 euthyroid HT patients and 119 healthy controls. Patients with HT had higher frequent consumption of animal foods (meat, fish, dairy products) than the control group, who showed higher intake frequencies of plant foods (legumes, fruits, vegetables and nuts). Moreover, volunteers from the control group more often preferred poultry over red and processed meat compared to HT patients. The study showed that meat consumption was associated with increased development of thyroid autoimmunity and the Mediterranean diet had a protective effect. The study demonstrated that markers of oxidative stress (i.e., AGEs) were significantly higher in HT subjects than in controls. The activities of glutathione peroxidase and thioredoxin reductase and total plasma antioxidant activity were lower, which indicates oxidative stress. They also demonstrated a significant dependence of oxidative stress parameters on the consumption of animal foods, especially meat. This study suggested a protective effect of a low intake of animal foods on thyroid autoimmunity and a positive influence on oxidant-antioxidant balance [141]. Moreover, Benveng et al. found an association between decreased serum thyroid autoantibody levels and the consumption of oily fish, which is rich in omega-3 fatty acids [147].

Giannakou et al. demonstrated that daily consumption of fruit and vegetables allows for the maintenance of low oxidative stress levels in HT patients. However, in their study, there was no significant association between TOS levels and the weekly consumption of meat, dairy products, alcohol, coffee or tea. The authors suggested that patients with HT should modify their diet to maintain BMI within the reference range [62].

Nutritional interventions in patients with HT focus on dietary restrictions (Table 3). Some studies demonstrated that lactose restriction could lead to decreased levels of TSH in HT patients. A study on patients with HT during LT4 therapy found that the level of TSH significantly decreased in the euthyroid and subclinical hypothyroid patients with lactose intolerance after an eight-week lactose-restricted diet. However, the level of fT4 did not significantly decrease after lactose restriction, which may be because fT4 levels in the studied patients were within the normal range from the beginning of the study or a relatively short follow-up period [148].

Few studies have been conducted on the usage of a gluten-free diet (GFD) in patients with HT. Some research suggested a relationship between gluten consumption and the development or progression of HT. A pilot study of euthyroid women with HT showed that GFD reduced thyroid antibody titers [149]. Another study by Velija et al. tested the response of subclinical hypothyroidism patients with HT treated with a gluten-free diet and Se supplementation in restoring a normal thyroid function. After six months, euthyroidism was restored in more patients who received Se and had a gluten-free diet compared to the control group who supplemented Se without any dietary intervention. Moreover, the reduction in serum TPOAb levels in the study group was significantly greater than in the control group. The results of this study suggested that the gluten-free diet together with Se supplementation is more effective compared to only Se supplementation in HT women with subclinical hypothyroidism [150]. Another study showed no reduction in thyroid autoimmunity after following a gluten-free diet. Pobłocki et al. conducted a randomized study with euthyroid women with HT receiving LT4. The control group’s diet contained gluten, and the study group was on a gluten-free diet for 12 months. During follow-up, there was a significant reduction in TSH levels in the study group [151]. Only a few studies suggest that gluten elimination may be helpful for some HT patients. It should be noted that this diet is very restrictive and difficult to follow and contributes to the risk of nutritional deficiencies. The studies conducted so far do not confirm that patients with HT should be on a gluten-free diet, therefore it is not recommended [152].

One study by Ostrowska et al. assessed the effectiveness of two reducing diets and their effect on thyroid parameters in female obese patients with HT. All women who received LT4, Se and zinc were randomly assigned to the study group following individually balanced elimination/reducing diets, in accordance with the previously performer food sensitivity tests, and the control group following reducing diets with the same caloric content, but without product elimination. The anthropometric and thyroid parameters have changed in both groups during the nutritional intervention. This research showed that weight reduction may improve thyroid function in patients suffering from obesity and HT. Moreover, an individually selected elimination reducing diet was more effective than classic reducing diets with the same energy intake and macronutrient content and can lead to better therapeutic outcomes, which may cause an anti-inflammatory effect [153].

One case report showed a novel approach that led to the improvement of symptoms and a reduction of thyroid antibodies in a 23-year-old woman with HT. The woman presented with symptoms of fatigue, hair loss, energy and mood disturbance, problems with insomnia and daytime napping. The thyroid antibodies were strongly positive, with a normal TSH level. Integrative treatment was started, which involved nutritional changes and micronutrient supplementation. This supplementation supported the methylation cycle, anti-oxidant capacity and stress management, and included vitamins C, B1, B2, B5, B6, Pyridoxal-5 Phosphate, zinc picolonate, L-5 methyltetrahydrofolate, magnesium glycinate, selenomethionine, N- Acetyl Cysteine and methyl B12. The patient followed a paleo-style diet without grains and dairy products and increased consumption of bone broth and fermented foods as well as organic animal protein as tolerated. In addition, daily meditation and mindfulness techniques were recommended, and gentle exercise three times a week was added. After 15 months of treatment, there was a reduction in antithyroid antibodies and a significant relief of symptoms. This case demonstrated the potential benefits of an integrative approach to autoimmunity and oxidative stress in HT [154].

In a pilot study by Abbott et al., women participated in a 10-week online health coaching program focused on implementing an “autoimmune protocol” diet. They applied a modified paleolithic diet. In the referred study, there were no significant changes in thyroid function markers, as well as serum antithyroid antibody concentrations, although the number of immune cells and an inflammatory processes marker (hsCRP) were decreased. These results suggest that an “autoimmune protocol” may decrease inflammation and modulate the immune system. Moreover, the therapy improves health-related quality of life (measured by 36-Item Short-Form Health Survey) and reduces symptoms of the diseases (measured by the Medical Symptoms Questionnaire) [155]. A case study with a 49-year-old obese HT woman indicated that a modified autoimmune paleo low-calorie diet might improve TSH, TPOAb, body composition and lipid profile [156].

An anti-inflammatory diet rich in vitamins, minerals and polyphenols is recommended as diet therapy for HT [11,21,152]. Natural antioxidants like vitamins A, C, and E are found in products of plant origin, including a wide variety of vegetables and fruits. Sources of vitamin C include broccoli, peppers, black currant, strawberries, lemons, spinach, kiwifruit, oranges, grapefruit, limes, tomatoes, raspberries, asparagus, pineapples, fennel and parsley. The best source of vitamin E is avocado, nuts, seeds, egg, milk and whole grains. In addition, vitamin A is present in foods such as liver, carrot, broccoli, butter, pumpkin, cheese, egg, mango and milk [157]. According to the current findings, the Mediterranean diet may show the most benefits for HT patients with its antioxidant properties [141].

**Table 3:** The summary of dietary interventional studies impact on the treatment and management of HT.

Characteristics of the Diet	Duration of the Study	Cohort Studied	Examined Parameters	Results	Ref.
**Lactose-restricted diet**	eight weeks	83 HT patients taking LT4: euthyroid (n = 53), subclinical (n = 19), overt hypothyroidism (n = 3), subclinical hyperthyroidism (n = 8)	Serum levels of TSH, fT4, calcium and parathormone, titers of TPOAb	- level of TSH significantly decreased in the euthyroid and subclinical hypothyroid patients with lactose intolerance following lactose restriction; - the level of TSH in the euthyroid patients without lactose intolerance remained unchanged; - the levels of PTH, fT4 and calcium did not significantly change after lactose restriction.	[148]
**Gluten-free diet**	six months	34 women with HT: *Study group* (n = 16): gluten-free diet; *Control group* (n = 18): without any dietary treatment	Serum titers of TPOAb and TgAb, levels of TSH, fT3, fT4 and 25(OH)D	- in the control group serum TSH, fT3, fT4 and 25(OH)D levels remained at the similar levels; - in the study group the gluten-free diet reduced serum titers of TPOAb and TgAb and slightly increased 25(OH)D levels; - no differences in TSH, fT3 and fT4 levels in the study group after gluten-free diet; - in the study group TPOAb titers correlated with 25(OH)D levels.	[149]
**Gluten-free diet with selenium supplementation**	six months	98 drug-naive HT women with subclinical hypothyroidism *Study group* (n = 50): 200 µg selenium and gluten-free diet *Control group* (n = 48)*:* selenium supplementation without any dietary treatment.	Serum titers of TPOAb and TgAb, levels of TSH, fT4 and fT3	- euthyroidism was restored in 74% of the study group and in 58.3% of the control group; - TSH, TPOAb and TgAb levels were significantly reduced in both groups; - serum TPOAb titer in the study group had a more significant decrease (by 49%) than those in the control group (by 34%).	[150]
**Gluten-free diet connected with a healthy lifestyle promotion and education by our expert dietitian**	12 months	62 euthyroid HT women with LT4 treatment: *Study group* (n = 31): gluten-free diet, healthy lifestyle education; *Control group* (n = 31): no changes in the diet, diet containing gluten	Serum levels of TSH, fT3, fT4, titers of TPOAb and TgAb, body weight and BMI	- a reduction in TSH levels after three, six and 12 months in the study group; - an increase in fT4 concentrations after six and 12 months; - no differences in TPOAb and TgAb, fT3 or fT4 levels in both groups after 12 months.	[151]
**Elimination/reducing diets with selenium and zinc supplementation** **1400–1600 kcal/day (with a deficit of about** **1000 kcal/day)** Macronutrient content: 25% protein, 30% fat, and 45% carbohydrate	six months	100 women previously diagnosed with HT, obesity and receiving L-thyroxine, 200 mcg of 1selenomethionine/day and 30 mg of zinc gluconate/day: *Study group:* 50 women following individually adjusted elimination/reducing diets, *Control group:* 50 women following reducing diets with the same caloric content (without ingredient elimination)	Serum levels of TSH, fT3, fT4, titers of TPOAb and TgAb, body weight and BMI	- the decrease in BMI, body fat percentage, TSH concentration, TPOAb and TgAb levels in the study group were significantly greater compared to the control group; - the study group showed significantly greater increases in fT4 and fT3 levels than the control group; - after six months there was a positive correlation between the difference in body fat content and TSH levels and between TPOAb levels and BMI and a negative correlation between serum fT4 or fT3 and BMI.	[153]
**Paleo-style diet without grains and dairy products with micronutrients supplementation**	15 months	A case study of 23-year-old euthyroid woman diagnosed eight months prior with HT	Serum levels of TSH, fT4, zinc, ferritin, vitamin D and B12, titers of TgAb and TPOAb	- a significant reduction in TPOAb and TgAb; - improvement in symptoms.	[154]
**Autoimmune Protocol Diet, Supported Lifestyle Intervention**	10 weeks	17 normal or overweight female subjects with a prior diagnosis of HT	Blood cell count, metabolic profile, levels of TSH, fT4, fT3, hsCRP, titers of TPOAb and TgAb, Short Form Health Survey, Medical Symptoms Questionnaire	- no statistically significant changes in TSH, fT4, fT3 and thyroid antibodies; - a statistically significant improvement in health-related quality of life; - the clinical symptom burden decreased significantly; - hsCRP significantly decrease by 29%.	[155]
**Modified auto-** **immune Paleo low-calorie diet (1200 kcal)**	six months	A case study of newly diagnosed 49-year-old obese woman with HT, medically free from any chronic diseases	levels of fT4, fT3, TSH, IFG, insulin, TG, non-HDL and HDL cholesterol titers of TPOAb, body composition, anthropometric measurements	- a significant reduction in body weight, BMI, waist and hip circumference, WHR, fat mass, levels of TC, TG, LDL and non-HDL cholesterol, insulin, IFG, TSH, and TPOAb; - fT3 and fT4 remained within normal reference range; - significant increase in HDL cholesterol level.	[156]

HT—Hashimoto’s thyroiditis; n—Number of participants; LT4—Levothyroxine; TPOAb—anti-thyroid peroxidase antibodies; TgAb—Anti-thyroglobulin antibodies; TSH—Thyrotropin; fT4—Free thyroxine; fT3—Free triiodothyronine; 25(OH)D—25-hydroxyvitamin D; IU—International unit; TC—Total cholesterol; TG—Triglycerides; HDL—High-density lipoprotein; hsCRP—High-sensitivity C-reactive protein; BMI—Body mass index; WHR—Waist-Hip Ratio.

### 6.2. Gut Microbiota in HT

The microbiota plays a significant role in maintaining nutritional, metabolic and immunologic homeostasis [158,159]. Evidence suggests that intestinal dysbiosis, bacterial overgrowth, and increased intestinal permeability promote the development of inflammatory and autoimmune diseases, including HT [160,161]. Cayres et al. analyzed 40 patients with HT and 53 controls. They showed an increase in the Bacteroides species and a decrease in Bifidobacterium in patients with HT. Furthermore, the Lactobacillus species were less frequent in patients who underwent thyroid hormones replacement therapy than those without LT4 treatment. This study also demonstrated significant differences in the consumption of vegetables, fruits, dairy products, carbohydrates, proteins and saturated fats between HT patients and the control group. They suggested that diet might have an important role in modulating the gut microbiota in patients with HT. Moreover, the concentration of zonulin increased, pointing to a leaky gut in patients with HT [161]. In a cross-sectional study with 45 HT patients with euthyroidism, 18 HT patients with hypothyroidism, and 34 healthy controls, the microbial richness and diversity of gut microbiota was significantly lower in patients with HT, especially in hypothyroidism, compared with the controls [162]. Another cross-sectional study of 28 HT patients and 16 matched healthy controls confirmed that HT patients have altered gut microbiota [163]. Therefore, further research should be carried out to show the role of the microbiota in the pathogenesis and progression of HT.

## 7. Conclusions

Many patients with HT, even in the euthyroid state, have high oxidative stress, excess body weight and metabolic disorders. Oxidative stress may be a significant risk factor in the pathogenesis and progression of HT and the development of complications. Moreover, the latest systematic review and meta-analysis showed that obesity is significantly associated with HT and high levels of TPOAb. Therefore, lifestyle changes, including body weight normalization, appropriate pharmacotherapy, along with adjusted nutrition and supplementation, are essential elements of medical care for patients with HT in improving health, life comfort and reducing the rate of complications. A few recent systematic reviews and meta-analyses confirmed significantly lower vitamin D levels in patients with HT compared to healthy subjects and suggested that vitamin D supplementation reduces TPOAb titers. However, in a majority of cases, no significant associations were found between serum vitamin D and TgAb, TSH, fT3, and fT4 concentrations. Some studies also indicated a beneficial role for Se supplementation, but there is insufficient evidence. So far, none of the European or American Endocrine/Thyroid Societies recommended Se supplementation in HT. Therefore, more studies are needed in this regard to show whether this observation is clinically relevant. Moreover, it was proved that excessive iodine intake is associated with a higher incidence of HT, while in areas with iodine deficiency, a lower prevalence has been demonstrated. The association between iron deficiency and thyroid autoimmunity has not been well established. A recent systematic review and meta-analysis demonstrated that iron deficiency significantly increases the risk of positive TPOAb and TgAb. In addition, many patients with HT are iron deficient due to comorbidities such as autoimmune gastritis, which causes a reduction in iron absorption or celiac disease, which leads to iron loss. We are uncertain that patients with HT had a higher risk of developing magnesium deficiency than healthy controls. More research is needed to evaluate the association between magnesium levels and HT. Pernicious anaemia caused by vitamin B12 deficiency often coexists with HT, as was proved in several studies, and therefore its addition to the diet is essential. Moreover, a proper diet that prevents nutritional deficiencies can improve the quality of life of HT patients. Results of the studies indicated that HT patients should receive routine medical checkups, including testing the vitamin and micronutrient status in the body. Additionally, HT patients should be educated about nutrition because a well-balanced diet is one of the most critical elements in preventing nutritional deficiencies. Furthermore, there are still no comprehensive studies investigating dietary habits and their influence on HT and the specific diet recommended for these patients. Recent studies suggest that an anti-inflammatory diet rich in vitamins and minerals and low in animal-based foods may be protective. There is insufficient evidence to support a gluten-free diet for all HT patients. The cooperation of endocrinologists, nutritionists and other specialists is essential for the comprehensive care of HT patients and the prevention of complications, including metabolic disorders. Nevertheless, more studies concerning the role of micronutrients and diet in the development and progression of HT are necessary.
